# Combining a machine-learning derived 4-lncRNA signature with AFP and TNM stages in predicting early recurrence of hepatocellular carcinoma

**DOI:** 10.1186/s12864-023-09194-8

**Published:** 2023-02-27

**Authors:** Yi Fu, Anfeng Si, Xindong Wei, Xinjie Lin, Yujie Ma, Huimin Qiu, Zhinan Guo, Yong Pan, Yiru Zhang, Xiaoni Kong, Shibo Li, Yanjun Shi, Hailong Wu

**Affiliations:** 1 Shanghai Key Laboratory of Molecular Imaging, Zhoupu Hospital, Shanghai University of Medicine and Health Sciences, Shanghai, China; 2 Collaborative Innovation Center for Biomedicines, Shanghai University of Medicine and Health Sciences, Shanghai, China; 3 School of Medical Instruments, Shanghai University of Medicine and Health Sciences, Shanghai, China; 4 Department of Surgical Oncology, Jinling Hospital, Medical School of Nanjing University, Nanjing, China; 5 Central Laboratory, Department of Liver Diseases, Shuguang Hospital, Shanghai University of Chinese Traditional Medicine, Shanghai, China; 6 School of Health Science and Engineering, University of Shanghai for Science and Technology, Shanghai, China; 7 School of Kinesiology, Shanghai University of Sport, Shanghai, China; 8 Department of Infectious Disease, Zhoushan Hospital, Wenzhou Medical University, Zhoushan, China; 9 Abdominal Transplantation Center, General Surgery, School of Medicine, Ruijin Hospital, Shanghai Jiao Tong University, Shanghai, China

**Keywords:** Long non-coding RNA signature, Hepatocellular carcinoma, Early recurrence, Machine learning

## Abstract

**Background:**

Near 70% of hepatocellular carcinoma (HCC) recurrence is early recurrence within 2-year post surgery. Long non-coding RNAs (lncRNAs) are intensively involved in HCC progression and serve as biomarkers for HCC prognosis. The aim of this study is to construct a lncRNA-based signature for predicting HCC early recurrence.

**Methods:**

Data of RNA expression and associated clinical information were accessed from The Cancer Genome Atlas Liver Hepatocellular Carcinoma (TCGA-LIHC) database. Recurrence associated differentially expressed lncRNAs (DELncs) were determined by three DEG methods and two survival analyses methods. DELncs involved in the signature were selected by three machine learning methods and multivariate Cox analysis. Additionally, the signature was validated in a cohort of HCC patients from an external source. In order to gain insight into the biological functions of this signature, gene sets enrichment analyses, immune infiltration analyses, as well as immune and drug therapy prediction analyses were conducted.

**Results:**

A 4-lncRNA signature consisting of AC108463.1, AF131217.1, CMB9-22P13.1, TMCC1-AS1 was constructed. Patients in the high-risk group showed significantly higher early recurrence rate compared to those in the low-risk group. Combination of the signature, AFP and TNM further improved the early HCC recurrence predictive performance. Several molecular pathways and gene sets associated with HCC pathogenesis are enriched in the high-risk group. Antitumor immune cells, such as activated B cell, type 1 T helper cell, natural killer cell and effective memory CD8 T cell are enriched in patients with low-risk HCCs. HCC patients in the low- and high-risk group had differential sensitivities to various antitumor drugs. Finally, predictive performance of this signature was validated in an external cohort of patients with HCC.

**Conclusion:**

Combined with TNM and AFP, the 4-lncRNA signature presents excellent predictability of HCC early recurrence.

**Supplementary Information:**

The online version contains supplementary material available at 10.1186/s12864-023-09194-8.

## Introduction

The recent global cancer statistics study indicated that the new cases and deaths of liver cancer were 905,677 and 830,187 respectively and rank sixth in terms of incidence and third in terms of mortality [1]. Approximately 75–85% of primary liver cancer cases are caused by hepatocellular carcinoma (HCC) [1]. Although the main risk factors of HCC show regional differences, chronic hepatitis B or C infection remains the major causes of HCC [2, 3]. Benefit from vaccination against HBV, the incidence of HCC in high-risk countries of Eastern Asia has been dramatically reduced [3]. However, incidence rates of HCC in regions like Europe, Northern and South America, Australia/New Zealand, which were low-risk regions display the opposite trend or remain at a high-level plateau [4]. Thus, the overall global burden of liver cancer is increasing over time.

Long non-coding RNAs (lncRNA), a class of non-coding transcripts that exceed 200 nucleotides in length, have been identified as important regulators in the development of various cancers [5, 6]. Cancer-related lncRNAs are involved in genomic instability, sustained proliferation, activation of invasion and metastasis, and cell death resistance in cancer cells by means of diverse mechanisms [7] through binding with RNA, DNA, protein or encoding small peptides [8–10]. For example, lncRNA GHET1 promoted HCC cell tumorigenesis by activating H3K27 acetylation and regulating ATF1 [11]. LINC01234, a potential prognostic or therapeutic HCC marker, could modulate aspartate metabolic reprogramming and promote HCC progression [12]. Cancer-associated fibroblasts secreted exosomal lncRNA TUG1 facilitated HCC cell glycolysis, migration, and invasion via miR-524-5p/SIX1 axis [13]. LncRNA DANCR promoted HCC stemness by regulating mRNA stabilization [14]. LncRNA PVT1 promotes HCC cell proliferation and stemness by stabilizing NOP2 [15]. Besides experimental evidences, more lncRNA-disease associations were elucidated by powerful bioinformatics tools and models, which could help to underlie disease mechanisms at the level of lncRNA and facilitate the detection of biomarkers for diagnosis and prognosis, as well as disease prevention and treatment [16, 17]. Therefore, several lncRNAs have been reported to serve as diagnosis and prognosis biomarkers for HCC. Lnc-PCDH9-13:1 was upregulated in HCC tissues, serum and saliva of the patients and could serve as a biomarker for detecting early HCC [18]. Circulating exosomal lncRNA-ATB was identified as an independent predictor HCC overall survival and disease progression [19]. A panel of serum circulating lncRNA LINC00153, UCA1 and AFP was reported to have satisfactory sensitivity and specificity for HCC diagnosis [20]. A signature consisting of 50 lncRNA pairs could serve as an independent powerful prognostic indicator for HCC overall survival prediction [21]. Moreover, lncRNA signatures associated with genome instability, macrophages [22, 23], pyroptosis, ferroptosis, tumor microenvironment, m6A regulator, autophagy, hypoxia, glycolysis and EMT [24–31] were established for predicting overall survival in HCC.

Nearly 70% HCC patients had postsurgical recurrence in 5 years. Postsurgical recurrence is the primary limitation for the improvement of HCC prognosis [32]. Clinically, a recurrence within two years of surgery is referred to an early recurrence while a recurrence after two years is called a late recurrence [33]. Earlier studies has indicated that near 70% of recurrence was early recurrence, and HCC patients with early recurrence had a significantly lower 5-year overall survival rate compared to HCC patients with late recurrence [34]. Therefore, construction of signature predicting HCC early recurrence would enable an improved surveillance strategy and prognosis.

In this study, we collected data from TCGA-LIHC databases to construct a 4-lncRNA prognostic signature for HCC early recurrence. Multivariate Cox regression, Kaplan-Meier, nomogram and ROC analyses were performed to evaluate the predictive potential of this signature. KEGG, GO, GSEA were performed to explore the underlying mechanism of HCC early recurrence. Intratumor immune infiltration status and drug response prediction analyses were used to evaluate the potential of this signature in predicting therapeutic responses. In addition, the significance of this signature was further validated in external HCC cohorts (Figure S1).

## Materials and methods

### Data mining for candidate recurrence related dysregulated lncRNAs in hepatocellular carcinoma

Liver cancer RNA expression data and associated clinical features were downloaded from The Cancer Genome Atlas Liver Hepatocellular Carcinoma (TCGA-LIHC) database (https://portal.gdc.cancer.gov/projects/TCGA-LIHC). The expression profile and clinical features of 314 HCC patients with complete overall survival (OS) and disease free survival (DFS) record were reserved after carefully screening. These 314 patients were then randomly divided into the training group (N = 157) and the validation group (N = 157) by R package “caret” [35]. Clinical features of these patients including OS, DFS, TNM stages, cirrhotic status, vascular invasion, AFP, race, gender and age have been summarized (Table S1). Next, the differentially expressed lncRNAs (DElncs) were analyzed between the training group (N = 157) and the non-tumor group (N = 50). Differentially expressed gene (DEG) analyses were conducted by R packages “DESeq2” [36], “edgeR” [37, 38] and “limma” [39] with the cut-off value of |log_2_FC| > 1 and FDR < 0.05. Venn plots were drawn to identify the common dysregulated lncRNAs in all three DEG methods by R package “VennDiagram” [40]. Batch DFS survival analyses of the above dysregulated lncRNAs in the training group were then performed by R package “survival” [41] with both log-rank [42] and cox [43] methods with a cut-off value of P < 0.05. Finally, 81 candidate recurrence related dysregulated lncRNAs were obtained from the intersection of two survival analyses methods.

### Construction and validation for lncRNA-based prognostic signature for HCC recurrence

Dimensionality reduction of candidate lncRNAs used for signature construction were further conducted by three different machine learning methods including least absolute shrinkage and selection operator (LASSO) [44], random forest [45] and Support Vector Machine Recursive Feature Elimination (SVM-RFE) [46] in the training group with 81 candidate recurrence related dysregulated lncRNAs and DFS. LASSO, random forest and SVM-RFE were conducted by using R package “glmnet” [47], “randomForest” [45], and “e1071” [48] separately. More specifically, the cut-off values were lambda.min for LASSO, 5-fold cross validation with min(error) and max(accuracy) for SVM-RFE and top 30 DFS related lncRNAs for random forest. Venn plot was then drawn in three machine learning methods to identify 11 lncRNAs. Next, multivariate cox analysis of these 11 lncRNAs was performed in the training group (N = 157) with DFS by R package “survival” and 4 lncRNAs (AC108463.1, AF131217.1, CMB9-22P13.1, TMCC1-AS1) with a cut-off value of P < 0.05 were finally obtained for signature construction. The coefficients of these 4 lncRNAs for signature construction were calculated by multivariate cox analysis in the training group with DFS, and the risk score (RS) for each patient was calculated by the formula $risk score= \sum coefficient \times expression \left(gene\right)$. Receiver Operating Characteristic (ROC) analysis was performed by R package “pROC” [49] to evaluate the performances of the 4-lncRNA signature and three clinical features including AFP, TNM, as well as vascular invasion for HCC recurrence in the training group. All HCC patients were then further divided into the low- and high-risk group by median risk score from the training group.

### Tissue samples and clinical information

A total number of 44 patients who had liver surgery and were diagnosed with HCC between October 2018 and December 2019 at Jinling Hospital were included. HCC and paracancerous tissues were collected and treated following the protocols (81YY-KYLL-19-05) approved by the Ethics Committee of Jinling Hospital and Shanghai University of Medicine and Health Sciences. In addition, clinical characteristics including age, gender, serum AFP, numbers of tumor, cirrhotic status, vascular invasion and T stage were collected or analyzed. The follow-up data of 24 patients with available overall survival and disease free survival record until March 2022 were enrolled for survival analyses (Table S5). All patients enrolled in this study signed the written informed consent.

### Validation of the lncRNA-based prognostic signatures for HCC early recurrence

Since HCC early recurrence was defined as recurrence within 2 years in previous studies, those patients with follow-up time less than 24 months and no recurrence were excluded for the following studies of the 4-lncRNA signature with HCC early relapse. The HCC patients were 112 in the training group, 111 in the validation group and 223 in the entire group from the TCGA-LIHC cohort. To validate the prediction performance of the 4-lncRNA signature for HCC early recurrence, early recurrence cumulative event, cumulative hazard and probability were compared between the low- and high-risk group by R package “survminer” [50] in the training, validation and entire group. Multivariate cox analyses were also conducted to study the independent roles of the 4-lncRNA signature along with clinical characteristics like AFP, TNM stages, vascular invasion, gender and age. ROC analyses further identified that a combination model of lncRNA-based signature with AFP/TNM had the best prognostic prediction for HCC early recurrence. Nomogram was generated with the 4-lncRNA signature risk score, AFP and TNM stages and their corresponding coefficients from multivariate cox analyses, and the calibration curves were drawn by R package “regplot” [51]. Since no public external HCC dataset was available, we did further validation in clinical HCC samples by detecting the expression of 4 lncRNAs in clinical collected tumor and paired paracancerous tissues. Total RNA from 44 paired HCC and paracancerous tissue samples were collected by Jinling Hospital. cDNA was synthesized from total RNA by using the reverse transcription kit ReverTra Ace® qPCR RT Master Mix with gDNA Remover (TOYOBO). Real-time PCR reaction was conducted in 20 µL solution with Takara CYBR Premix Ex TaqII (Takara) in ABI BiosystemsTM 7500 Real-Time qPCR System (Applied Biosystems) by following the manufacturer’s protocol. Primers for AC108463.1, AF131217.1, CMB9-22P13.1, TMCC1-AS1 and 18s (Table S2) were purchased from GENEWIZ. To calculate the relative expression of lncRNAs, qRT-PCR results were interpreted by 2^−ΔΔCT^ method with 18s as the housekeeping gene. Furthermore, 24 HCC patients from Jinling cohort were enrolled for early recurrence analysis following the same criteria of TCGA-LIHC cohort.

### Comprehensive functional analyses of the 4-lnRNA prognostic signature

Total 223 HCC patients were divided into the low- and high-risk group by setting the 4-lncRNA prognostic signature median risk score as the cut-off. DEG analysis was performed between mRNA expression from the low- and high risk group, and all mRNAs were then arranged by descending |log_2_FC| for functional analyses. Kyoto Encyclopedia of Genes and Genomes (KEGG) [52], Gene Ontology (GO) and Gene Set Enrichment Analysis (GSEA) analyses were conducted by R package “clusterProfiler” [53]. The enriched KEGG pathways were determined by a cut-off value of |NES| > 1 and P < 0.05. The enriched GO terms included biological pathways (BP), cellular components (CC) and molecular functions (MF) were analyzed based on mRNAs with |logFC| > 1 and determined by a cut-off value of P < 0.05. GSEA analysis was performed with MSigDb C2: curated gene sets [54, 55] and enriched GSEA gene sets were determined by a cut-off value of |NES| > 1.5 and P < 0.05.

### Immune infiltration and clinical treatment response analyses

Single sample Gene Set Enrichment Analysis (ssGSEA) was chosen for studying immune infiltration with R package “GSVA” [56] and normalized enrichment score (NES) was calculated for 28 immune cell types in each 223 HCC samples. The NES of 28 immune cell types were compared between the low- and the high-risk group, and the correlation between the 4-lncRNA signature risk score and cells NES score was conducted by R function “cor.test”. Immune therapy response prediction was conducted with Tumor Immune Dysfunction and Exclusion (TIDE) algorithm [57, 58] and SubMap modules of GenePattern [59] by mapping with a public dataset of immunotherapy responses of 47 melanoma patients [60]. Drug response prediction was performed by R package “pRRophetic” [61] with Genomics of Drug Sensitivity in Cancer (GDSC) pharmacogenomics database [60, 62]. Ridge regression was used for estimating the half maximal inhibitory concentration (IC_50_) and 10-fold cross validation was used for predicting the accuracy.

### Statistical analyses

The sensitivity and specificity between two ROC curves were compared by DeLong’s test. The differences between Kaplan-Meier curves, cumulative hazard and events curves of survival analyses between two groups were compared by log-rank test. Univariate and multivariate analyses were conducted with the cox proportional hazards regression model. The comparisons of immune cells NES, drug IC_50_ and between the two groups, as well as the expression of lncRNAs between tumor and paracancerous tissue samples were analyzed by Wilcoxon test. The cut-off value of P < 0.05 was used for statistical significance.

## Results

### Identification of recurrence related lncRNAs

**Fig. 1:**
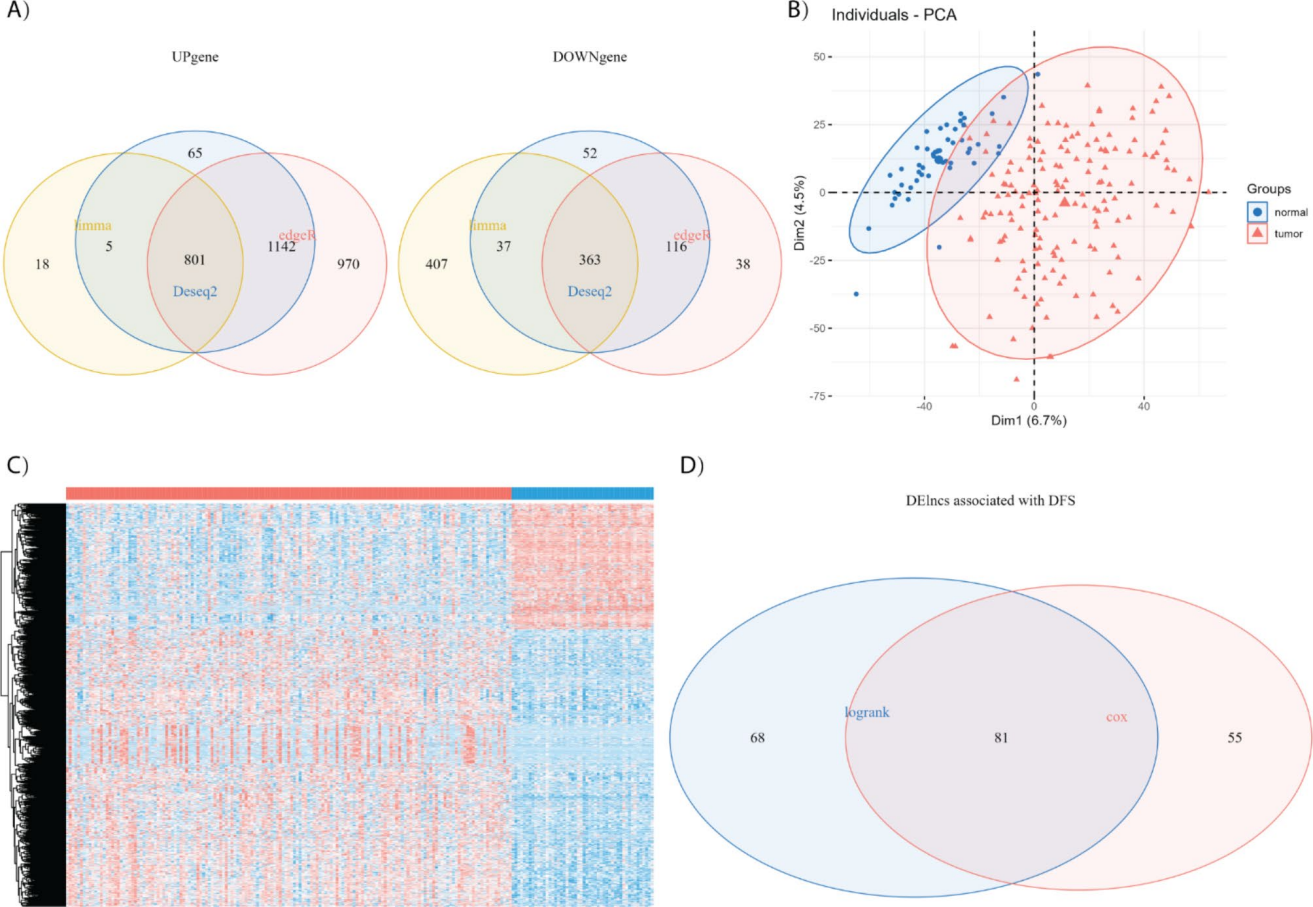
Identification of recurrence related dysregulated lncRNAs. (A) Venn plot of dysregulated lncRNAs in three different DEG analyses methods including DESeq2, edgeR and limma. Total 1164 dysregulated common lncRNAs (801 upregulated and 363 downregulated) were selected in TCGA training group; (B) PCA plot of 50 normal samples and 157 HCC samples of the training group based on the 1164 dysregulated common lncRNAs; (C) Heatmap of 1164 dysregulated common lncRNAs in 50 normal samples and 157 HCC samples of the training group; (D) Venn plot of recurrence related dysregulated lncRNAs from log-rank test and Cox regression survival analyses. There were 81 common DElncs related with HCC recurrence

To construct a lncRNA signature to predict postsurgical recurrence of HCC, we started to identify dysregulated lncRNAs in the TCGA training group. According to the sequences of 16,193 annotated human lncRNAs in GENCODE V30, we collected 10,795 lncRNAs for DEG analysis after excluding those with extremely low expression. Three methods, DESeq2, edgeR and limma-voom, were employed to identify differentially expressed lncRNAs (DElncs) between the HCC samples of the training group (N = 157) and the liver tissues of the normal control group (N = 50) with cut-off value of |log_2_FC| > 1 and FDR < 0.05. Compared with normal controls, 2581 (2013 upregulated and 568 downregulated), 3430 (2913 upregulated and 517 downregulated) and 1631 (824 upregulated and 807 downregulated) DElncs were determined respectively (Figure S2). Venn diagram analysis revealed 1164 (801 upregulated and 363 downregulated) common DElncs (Fig. 1A). The PCA plot and heatmap generated by the 1164 common DElncs could clearly distinguish HCCs from normal controls (Fig. 1B and C), suggesting that the 1164 common DElncs might closely associate with the onset and development of HCC. To identify recurrence associated DElncs, the log-rank test and Cox regression analysis were performed in the training group to evaluate disease free survival (DFS). After combining the candidates from the log-rank test (149 DElncs) and Cox regression analysis (136 DElncs), 81 common DElncs were identified as recurrence related lncRNAs (Fig. 1D).

### Construction of a 4-lncRNA prognostic signature for HCC recurrence

**Fig. 2:**
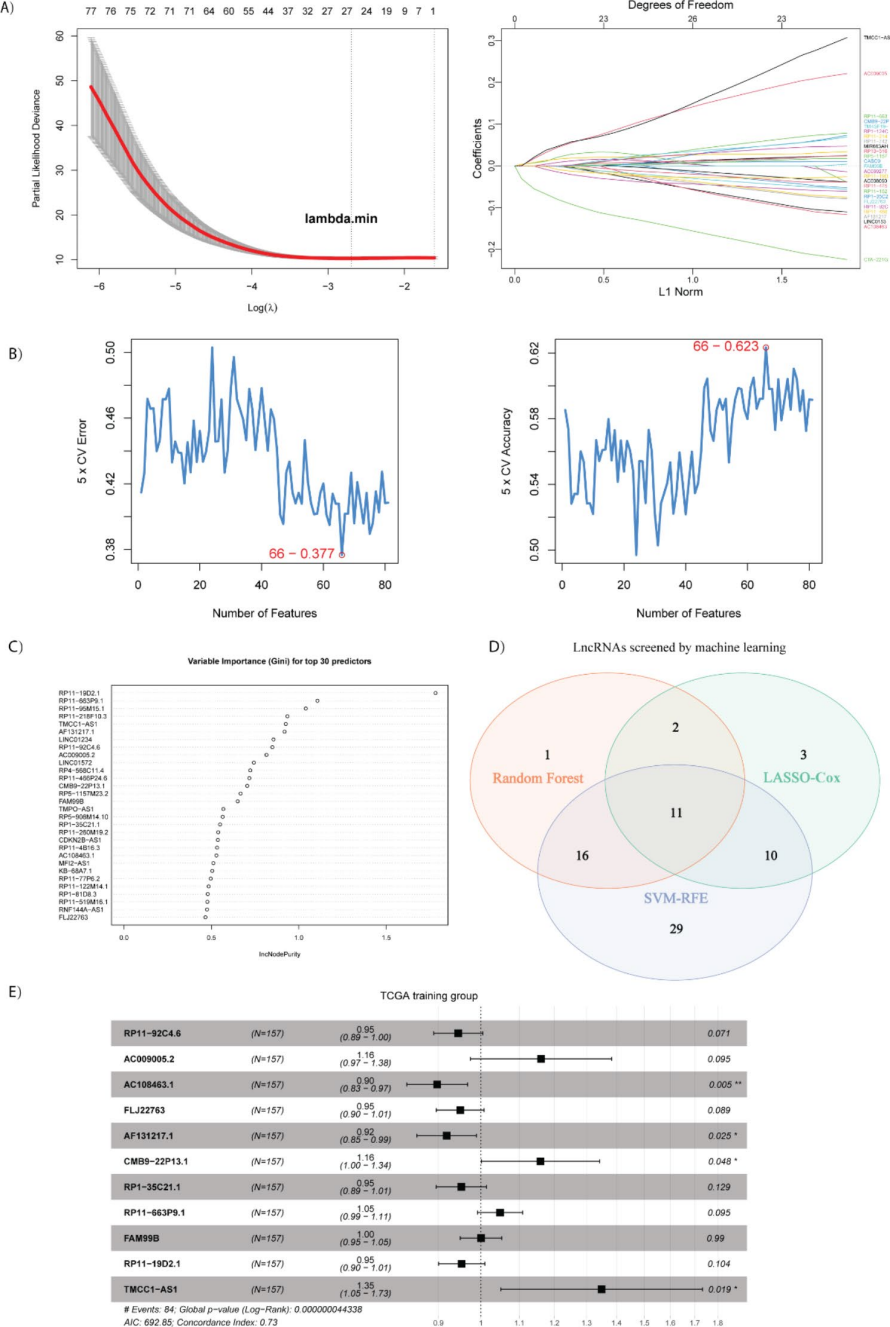
Candidate lncRNAs selection by survival analyses including LASSO, SVM-RFE and random forest. (A) Results of LASSO analysis, 26 lncRNAs were determined by lambda.min; (B) Results of SVM-RFE analysis, 66 lncRNAs were determined by 5-fold cross validation with min(error) and max(accuracy); (C) Top 30 lncRNAs related with disease free survival from random forest analysis; (D) Venn plot of selected lncRNAs from LASSO, SVM-RFE and random forest analyses, 11 lncRNAs were reserved for signature construction; (E) Multivariate cox analysis of 11 candidate lncRNAs with disease free survival in the training group, AC108463.1, AF131217.1, CMB9-22P13.1 and TMCC1-AS1 were independent risk factors for DFS

Based on the 81 recurrence associated lncRNAs, we then employed three classic machine learning methods, LASSO, Random Forest and SVM-RFE, to select important DElncs for predicting DFS in the training group. The LASSO, SVM-REF and Random Forest analyses selected 26, 66 and 30 candidates respectively (Fig. 2A to C). Venn diagram analysis collected 11 common Delncs for further analysis (Fig. 2D). Multivariate cox analysis of the 11 DElncs in the training group showed that 4 DElncs, AC108463.1, AF131217.1, CMB9-22P13.1, and TMCC1-AS1, are independent risk factors of DFS (Fig. 2E). We then constructed a prognostic signature based on the 4 DElncs, and calculated the risk score of individual HCC patients according to the linear combination of the regression coefficients and expression values of each DElncs [63]. Risk Score = (-0.0918*exp[AC108463.1]) + (-0.1112*exp[AF131217.1]) + (0.1484*exp[CMB9-22P13.1]) + (0.3737*exp[TMCC1-AS1]) (Table 1).

**Table 1:** The 4 DFS associated dysregulated LncRNAs in the training group patients from TCGA (N = 157)

Ensembl	Gene Symbol	P value	Hazard Ratio^a^	Coefficient^b^	associated diseases	Description
ENSG00000230499	AC108463.1	0.011	0.91	-0.0918	gastric cancer [89]	novel transcript
ENSG00000232855	AF131217.1	0.001	0.89	-0.1112	atherosclerosis [88]	novel transcript
ENSG00000173727	CMB9-22P13.1	0.011	1.16	0.1484	lung squamous cell carcinoma, breast cancer, hepatocellular carcinoma [90–93]	Finkel-Biskis-Reilly murine sarcoma virus (FBR-MuSV) ubiquitously expressed (FAU) pseudogene
ENSG00000271270	TMCC1-AS1	0.001	1.45	0.3737	hepatocellular carcinoma [94–97]	TMCC1 divergent transcript

^a, b^ Derived from the univariable Cox proportional hazards regression analysis

### The 4-lncRNA prognostic signature predicts HCC early recurrence

**Fig. 3:**
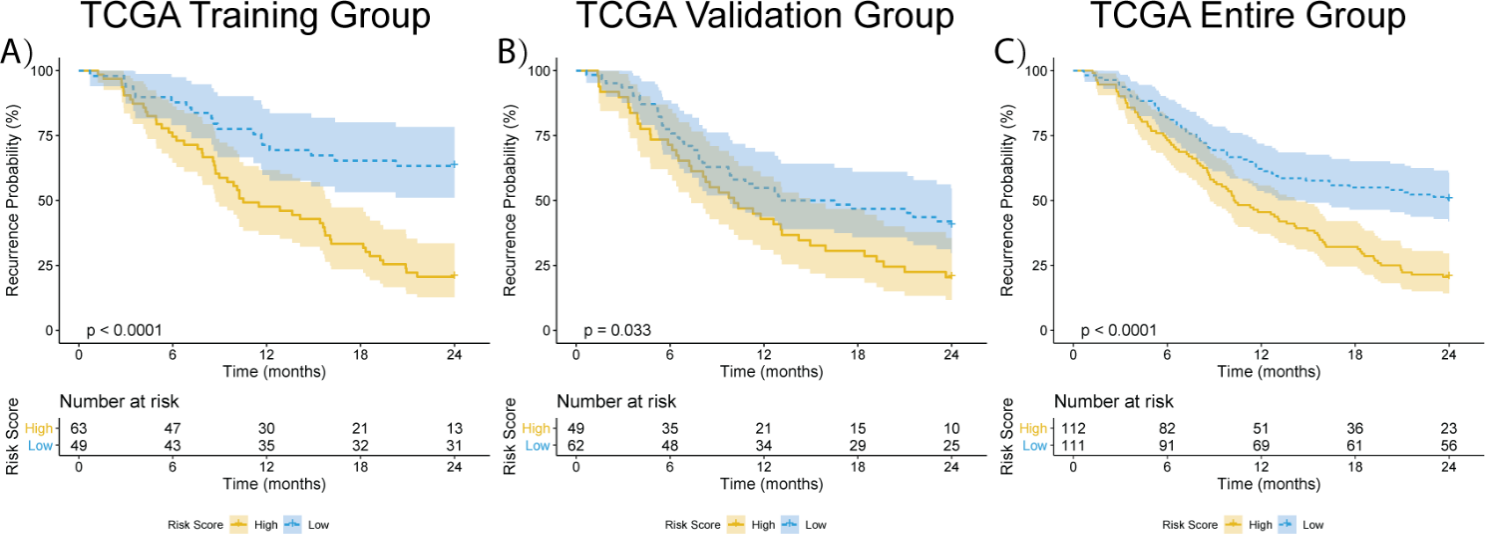
2-year DFS Kaplan-Meier curves of HCC patients. (A) 2-year DFS Kaplan-Meier curve in the training group (N = 112), the recurrence probability was higher in the high-risk group than that in the low-risk group (P < 0.0001); (B) 2-year DFS Kaplan-Meier curve in the validation group (N = 111), the recurrence probability was higher in the high-risk group than that in the low-risk group (P = 0.033); (C) 2-year DFS Kaplan-Meier curve in the entire TCGA group (N = 223), the recurrence probability was higher in the high-risk group than that in the low-risk group (P < 0.0001). Statistical significance was tested by the Log-rank method

Since HCC patients’ poor survival is largely attributed to early recurrence within two years after surgery, we intended to investigate whether the 4-lncRNA signature could provide a prognostic indication of HCC patients’ early recurrence. After excluding the patients whose follow-up data were collected less than 2-year and without recurrence records, 112 and 111 HCC patients were reserved in the training and the validation groups respectively. By setting the median risk score as a cut-off, HCC patients were categorized into the low- and high-risk groups. Kaplan-Meier survival analyses demonstrated that HCC patients from the high-risk group had shorter 2-year DFS in the training group (Fig. 3A, P < 0.0001), the validation group (Fig. 3B, P = 0.033) and the entire group (Fig. 3C, P < 0.0001). The HCC early recurrence rates in the high-risk group were up to 79% from all three groups (Fig. 3). In addition, cumulative event and cumulative hazard of the low-group patients were also compared to those of high-risk group patients. HCC patients in the high-risk group showed the higher cumulative events and cumulative hazard from all three groups (Figure S3). Thus, the findings by survival analyses indicate that the 4-lncRNA prognostic signature could predict HCC early recurrence within 2 years.

### Combination of the 4-lncRNA signature risk score with AFP and TNM improves the prognostic performance for HCC early recurrence

**Fig. 4:**
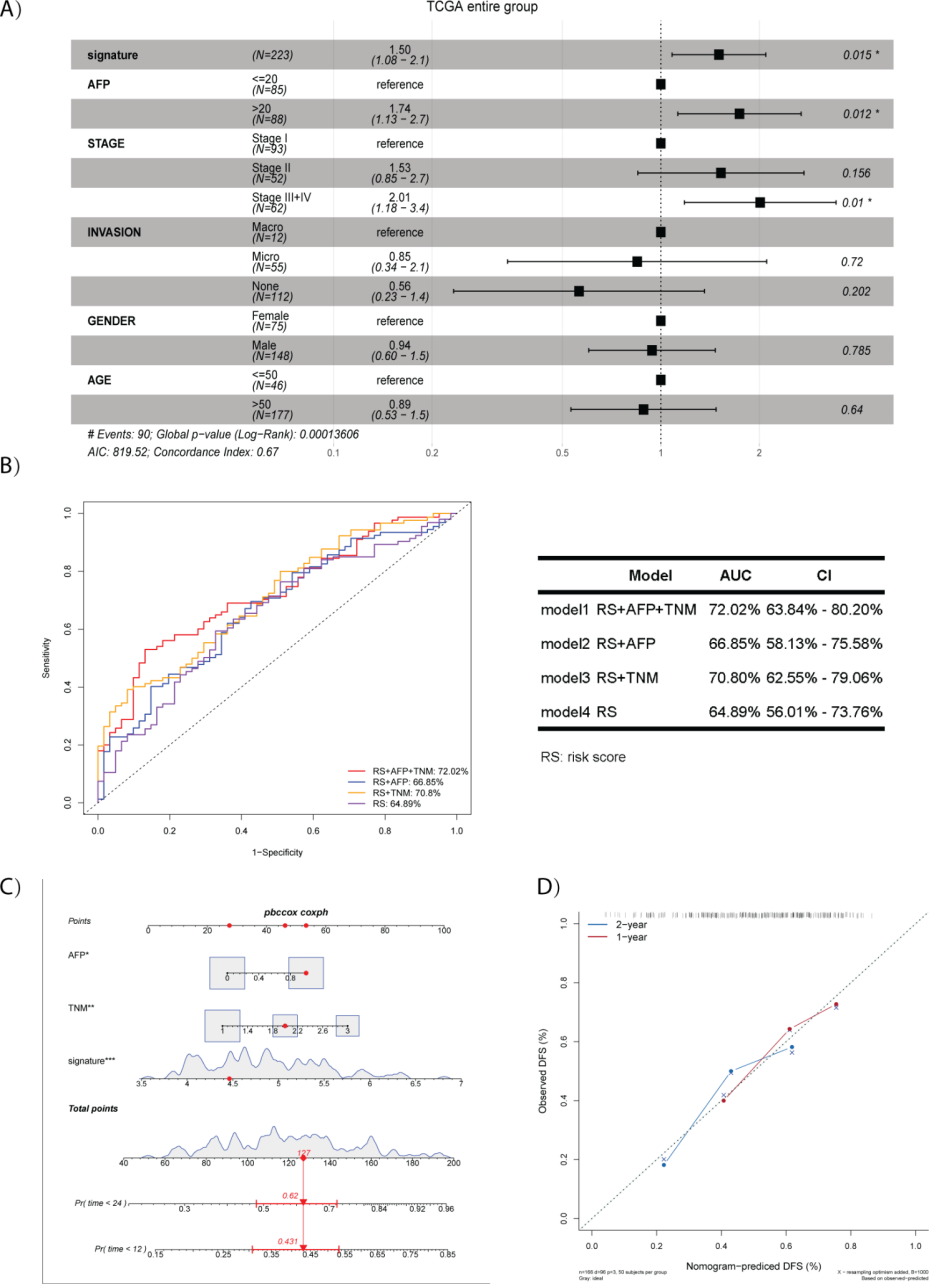
The combinations of the 4-lncRNA signature risk with clinical features. (A) Multivariate cox analysis of the 4-lncRNA signature risk score and clinical features with 2-year DFS in the entire group (N = 223). 4-lncRNA signature risk score, AFP and TNM stages are independent risk indicators for 2-year DFS (P < 0.05); (B) ROC analyses of model 1-4 with 2-year DFS. Model 1 (the combination of the 4-lncRNA signature risk score with AFP and TNM stages) had the largest AUC (72.02%) in all combined models; (C) Nomogram consisted of 4-lncRNA signature risk score, AFP and TNM stages for 1-year and 2-year DFS; (D) Calibration curves for integrated 4-lncRNA signature with AFP and TNM stages for 1-year DFS and 2-year DFS. RS: the 4-lncRNA signature risk score

To further evaluate the prognostic value of the 4-lncRNA signature, multivariate cox analyses of the risk score together with selected clinical features, including age, gender, AFP level, TNM stage and vascular invasion, were conducted in all 223 HCC patients with 2-year DFS. As shown in Fig. 4A, multivariate cox analyses suggest that the risk score (HR = 1.5, P = 0.015), AFP (HR = 1.74, P = 0.012) and TNM (HR = 2.01, P = 0.01 for stage III + IV) were independent risk indicators of HCC early recurrence. ROC analyses were then used for determining whether the combination of the independent risk indicators could improve prognostic performance. As shown in Fig. 4B, the combination of risk score with AFP and TNM showed the largest AUC (72.02%) for HCC early recurrence compared to risk score alone (AUC: 64.89%), risk score + AFP (AUC: 66.85%), and risk score + TNM (AUC: 70.80%). Meanwhile, the signature risk score, AFP and TMN stages were selected to establish a nomogram (Fig. 4C). The C-index for 1-year and 2-year DFS of this nomogram were 0.643 and 0.647, respectively. Moreover, a calibration curve revealed that the nomogram was good at predicting 1-year and 2-year DFS (Fig. 4D).

### Enriched KEGG pathways, GO terms and GSEA gene sets in the low- and high-risk groups

**Fig. 5:**
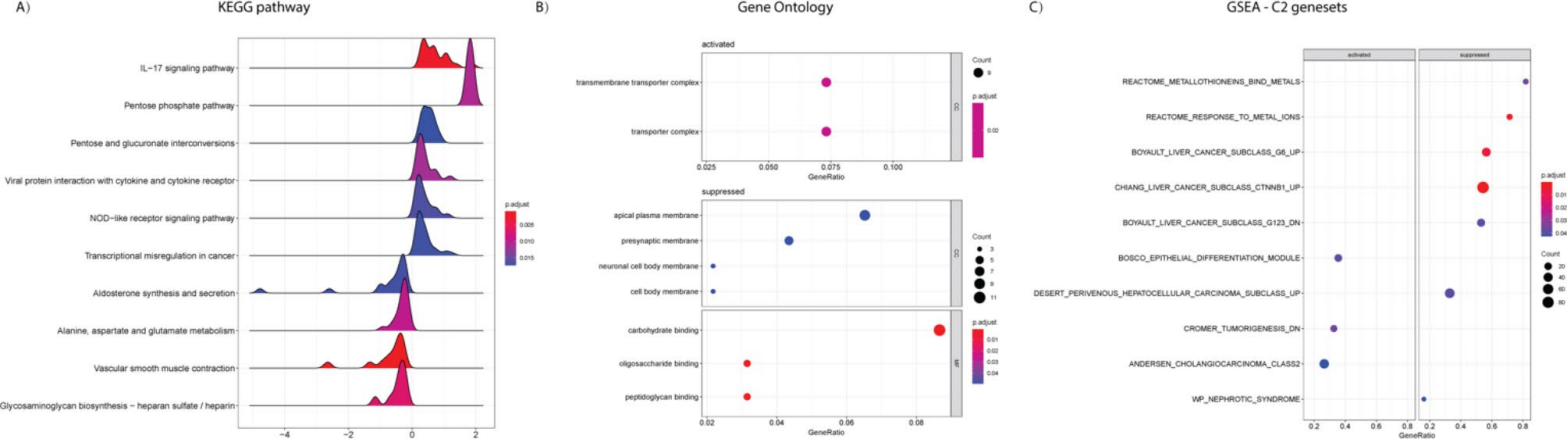
Enriched KEGG pathways, GO terms and GSEA gene sets in the low- and high-risk groups. (A) Top 6 activated (upper 6) and top 4 suppressed (lower 4) KEGG pathways enriched in the high-risk group; (B) GO terms activated and suppressed in the high-risk group. BP: biological function, CC: cellular component, MF: molecular function; (C) GSEA C2 gene sets activated and suppressed in the high-risk group

To further understand the functional differences between the low- and high-risk groups, KEGG, GO and GSEA analyses were conducted based on the differences of mRNA expression between the two groups. The representative pathways activated in the high-risk group included “IL-17 signaling pathway”, “Pentose phosphate pathway”, “Pentose and glucuronate interconversions”, “Viral protein interaction with cytokine and cytokine receptor”, “NOD-like receptor signaling pathway” and “Transcriptional misregulation in cancer”, while the pathways suppressed in the high-risk group included “Aldosterone synthesis and secretion”, “Alanine, aspartate and glutamate metabolism”, “Vascular smooth muscle contraction” and “Glycosaminoglycan biosynthesis - heparan sulfate/ heparin” (Fig. 5A). Several GO terms from biological process (BP), cellular component (CC) and molecular function (MF) were also significantly activated or suppressed in the high-risk group. For example, transporter complex was activated in the high-risk group, while apical plasma membrane, presynaptic membrane and carbohydrate binding were suppressed in the high-risk group (Fig. 5B). Besides, GSEA analysis with C2 gene sets revealed activated and suppressed gene sets in the high-risk group (Fig. 5C). The most significantly activated gene sets in the high-risk group were “BOSCO_EPITHELIAL_DIFFERENTIATION_MODULE”, “CROMER_TUMORIGENESIS_DN” and “ANDERSEN_CHOLANGIOCARCINOMA_CLASS2”, while the most significantly suppressed gene sets in the high-risk group were “REACTOME_METALLOTHIONEINS_BIND_METALS”, “REACTOME_RESPONSE_TO_METAL_IONS”, “BOYAULT_LIVER_CANCER_SUBCLASS_G6_UP”, “CHIANG_LIVER_CANCER_SUBCLASS_CTNNB1_UP”, “BOYAULT_LIVER_CANCER_SUBCLASS_G123_DN” and “DESERT_PERIVENOUS_HEPATOCELLULAR_CARCINOMA_SUBCLASS_UP”.

### Therapeutic responses prediction by the 4-lncRNA signature

**Fig. 6:**
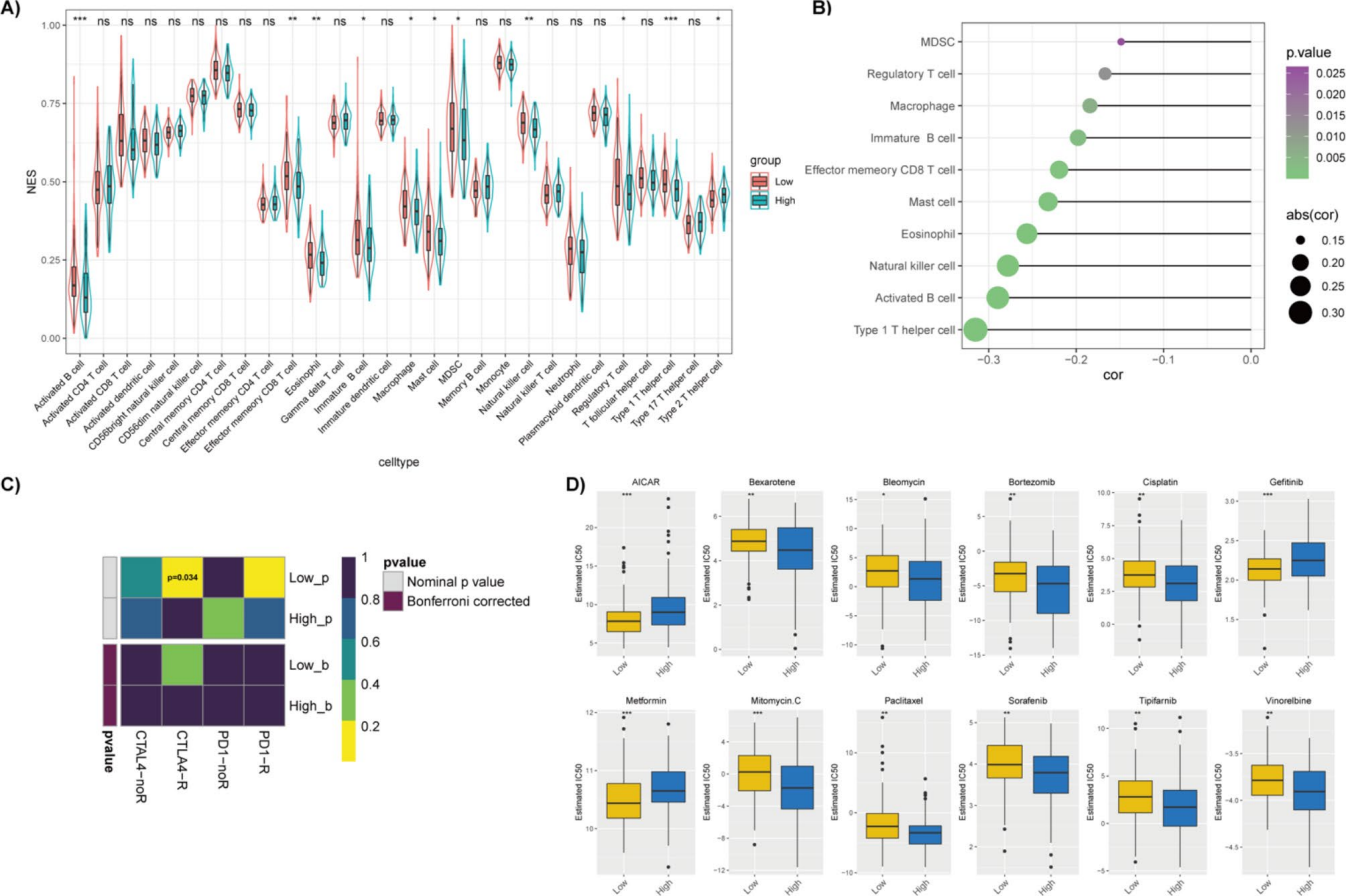
Immune infiltration analyses and clinical therapy response prediction of HCC patients in the low- and high-risk groups. (A) Immune cells NES comparisons between the low- and high-risk groups, 10 immune cells showed greater NES in the low-risk group (P < 0.05) and 1 immune cell showed greater NES in the high-risk group (P < 0.05); (B) Correlation between risk scores and immune cells NES, 10 immune cells were negatively related to risk scores (P < 0.05); (C) SubMap analysis of CTLA4 and PD-1 targeted immunotherapy in the low- and high-risk groups; (D) The predicted IC_50_ of clinical drugs in the low- and high-risk groups. The low-risk group patients had a lower IC_50_ in AICAR, gefitinib and metformin, while the high-risk group patients had a lower IC50 in bexarotene, bleomycin, bortezomib, cisplatin, mitomycin C, paclitaxel, sorafenib, tipifarnib and vinorelbine (P < 0.05 by Wilcoxon test)

Immunotherapy, as a novel treatment approach, has shown advantages in improving OS and DFS in HCC patients [64]. To investigate the potential of the 4-lncRNA signature in immunotherapy response prediction, the immune infiltration were compared between the low- and high-risk groups by calculating the NES of 28 immune cell types with ssGSEA. As shown in Fig. 6A, the intratumor infiltration of 10 immune cell types, including activated B cells, effector memory CD8 T cells, eosinophils, immature B cells, macrophages, mast cells, myeloid-derived suppressor cells, natural killer cells, regulatory T cells and type 1 T help cells, showed significantly higher NES in the low-risk group, whereas type 2 T help cells had significantly higher NES in the high-risk group. Correlation analyses illustrated that the NES of the above 10 immune cell types were negatively associated with risk scores (P < 0.05, Fig. 6B). Type 1 T helper cell, activated B cell and natural killer cell ranked the top3 negatively associated tumor infiltration lymphocytes (TILs) among them (|NES| > 0.25). However, although HCCs in the low-risk group showed greater immune cell infiltration, both TIDE predication and Submap analyses failed to show significant response advantages to anti-CTLA4 and anti-PD1 immunotherapy in the low-risk group (Fig. 6C). In addition, drug response prediction analysis indicated that the low-risk group HCC patients and high-risk group HCC patients showed significant differential responses to 27 drugs (Table S4). The low-risk group HCC patients may be more sensitive to AICAR, gefitinib and metforminin treatment, whereas the high-risk group HCC patients may better response to bexarotene, bleomycin, bortezomib, cisplatin, mitomycin C, paclitaxel, sorafenib, tipifarnib and vinorelbine (Fig. 6D). Thus, these findings indicate that the 4-lncRNA signature might act as a potential drug predictor.

### Validation of the 4-lncRNAs prognostic signature in clinical HCC samples

**Fig. 7:**
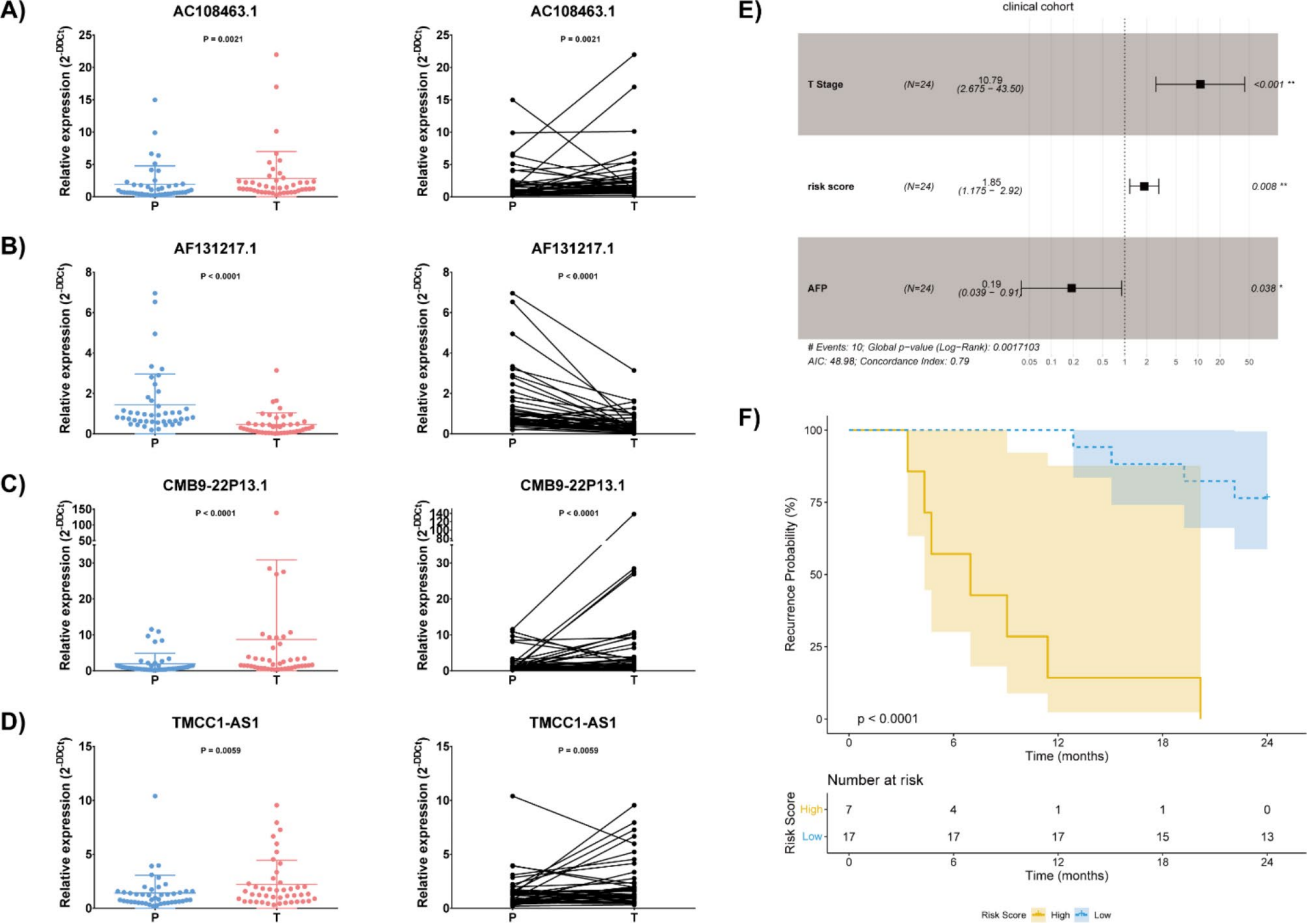
The relative expression of 4 lncRNAs in clinical HCC and paracancerous tissue samples (N = 44). (A) The expression of AC108463.1 was higher in HCC samples compared to paired paracancerous samples (P = 0.0021); (B) The expression of AF131217.1 was lower in HCC samples compared to paired paracancerous samples (P < 0.0001); (C) The expression of CMB9-22P13.1 was higher in HCC samples compared to paired paracancerous samples (P < 0.0001); (D) The expression of TMCC1-AS1 was higher in HCC samples compared to paired paracancerous samples (P = 0.0059). Statistical comparison was calculated by Wilcoxon test. (E) Multivariate cox analysis of the 4-lncRNA signature risk score, T stage and AFP with 2-year DFS in an external clinical cohort (N = 24). The 4-lncRNA signature risk score, T stage and AFP are independent risk indicators for 2-year DFS (P < 0.05); (F) 2-year DFS Kaplan-Meier curve in the clinical cohort (N = 24), the recurrence probability was higher in the high-risk group HCC patients than that in the low-risk group HCC patients (P < 0.0001). The significance was compared by log-rank test

Additionally, the expressions of these 4 lncRNAs were measured in a cohort of 44 paired HCC and their adjacent tissue samples. In line with our findings from TCGA-LIHC, AC108463.1, CMB9-22P13.1 and TMCC1-AS1 are highly expressed, and AF131217.1 is suppressed in HCC tissues compared to matched normal tissues (Fig. 7A-D). Furthermore, multivariate Cox analysis was conducted in 24 HCC patients whose 2-year DFS information was recorded. Results revealed that the 4-lncRNA signature risk score, T stage and AFP were independent risk factors for HCC early recurrence (Fig. 7E). Moreover, the 24 HCC patients were classified into low- and high-risk groups based on the combined model of 4-lncRNA signature risk score with AFP and T stage, Kaplan-Meier analysis illustrated that patients in the high-risk group had significantly poorer DFS within 2 years compared to those in the low-risk group (Fig. 7F, P < 0.0001).

## Discussion

In the current study, we developed a novel 4-lncRNA prognostic signature for early recurrence prediction in HCC by combining multiple DEG analysis methods, survival analysis methods and machine learning methods. This 4-lncRNA signature could fairly predict the early recurrence of HCC in TCGA-LIHC cohort, and the prediction performance could be further improved by the combination of the 4-lncRNA signature with TNM stages and AFP. According to the risk scores derived from the signature, HCC patients could be categorized into low- and high-risk groups. Functional analyses including KEGG, GO and GSEA were conducted to reveal the underlying mechanisms for HCC early recurrence. Moreover, immune infiltration analysis was employed to find out the immune microenvironment differences between the two groups, and prediction analyses of immune therapy and drug response provided useful information for differential clinical treatment. Finally, the prognostic performance of this 4-lncRNA signature was evaluated in an external HCC cohort.

DESeq2, edgeR and limma-voom are three popularly adopted approaches for DEG analysis [65]. DESeq2 uses shrinkage estimators for dispersion and fold change [36], edgeR adopts a Poisson super dispersion model for account for both biological and technical variability [37], and limma-voom is based on the linear model [66]. In the current study, we employed all those three statistical methods with the same fold for detecting differential expressed lncRNAs (DElncs) between the HCC samples (N = 157) and normal samples (N = 50). The DEG analyses results showed that edgeR found the most DElncs (3430), while limma-voom found the least DElncs (1631). The purpose of time-to-event survival analysis was to find out the DElncs associated with disease free survival. We imported two common survival analyses methods, log-rank and cox, and determined 81 DFS related DElncs [67]. Log-rank test is a non-parametric test for comparing the differences in survival between groups of patients [42], while cox proportional-hazards model is a semiparametric regression model for investigating the impact of variables on patient’s survival [43]. Further dimensionality reduction of DFS related DElncs was conducted by three different machine learning methods including LASSO, random forest and SVM-RFE. LASSO is a method for estimation in liner models with favorable properties of both subset selection and ridge regression [44]. Random forest constructs regression trees in the way of using the best among a subset of predictors randomly chosen at each node to be split [45]. SVM is a popular tool for nonlinear classification, regression and outlier detection [68], and SVM-RFE uses the weight magnitude as ranking criterion [46]. All these three machine learning methods have been universally used in gene selection with their advantages [69–74]. For instance, Xiao et al. utilized random forest and SVM for screening prognostic gene in malignant pleural mesothelioma [71], Shen et al. applied SVM for evaluating selective mutant genes and constructing a model for predicting HCC DFS [70], Xiao et al. used SVM-RFE and LASSO for identify candidate hub genes related to colorectal cancer [72]. Our group had also developed a 25-lncRNA signature for predicting the early recurrence of HCC patients by LASSO, but 25 lncRNAs are too many for further validation and clinical application [75]. Considering the individual advantage of LASSO, random forest and SVM-RFE, we have chosen these three popular machine learning methods for feature gene selection in this study.

Kaplan-Meier plot confirmed that the 4-lncRNA signature could successfully classified HCC patients into the low- and high-risk groups and predict early recurrence. Several previous developed lncRNA-based signatures were reported for HCC survival prediction and showed better performance than clinicopathological factors [76–80]. For example, a 3-lncRNA signature by Gu et al. could well predict both recurrence free survival and overall survival in small HCC patients [76], a 15-lncRNA classifier by Zhang et al. effectively identified HCC recurrence [77], a 7-lncRNA classifier by Lv et al. predict early recurrence within 2 years after surgical resection for HCC [78]. The prognostic performance of this 4-lncRNA signature is comparable to other developed lncRNA-based signatures [76–80], while the differences of selected lncRNAs in each lncRNA-based signature might be due to the specific feature gene selection strategy and different training set. Moreover, the combined model of the 4-lncRNA signature risk score, AFP and TNM stages further improve the 2-year DFS prediction with an AUC of 72.02%. Functional analyses were performed to explore the differences between the low- and high-risk groups. Some KEGG pathways activated in the high-risk group are favorable in HCC pathogenesis. For example, IL-17 was reported to promote hepatocellular carcinoma progression [81, 82], the pentose phosphate pathway (PPP) is one of the essential components of cellular metabolism and plays a key role in HCC [83, 84]. Immune infiltration analyses were performed with ssGSEA to elucidate the heterogeneous immune environment in the low- and high-risk group HCC patients. More immune cells had higher NES in the low-risk group and were negatively associated with the 4-lncRNA signature risk score. In addition, the top3 TILs which are negatively related to risk score, Type 1 T helper cell, activated B cell and natural killer cell, are well-known antitumor immune cells participated in cancer immune therapy process [85–87], further indicating that this 4-lncRNA signature could potentially predict HCC patients’ prognosis after surgery. Although this signature failed in predicting the response to cancer immunotherapy of HCC patients, drug response prediction suggested that the low-risk group patients are more sensitive to AICAR, gefitinib and metformin treatments, whereas the high-risk group patients are more sensitive to bexarotene, bleomycin, bortezomib, cisplatin, mitomycin C, paclitaxel, sorafenib, tipifarnib and vinorelbine.

In this study, the expression regulation of the 4 lncRNAs has been validated in an external HCC cohort containing 44 paired tumor and matched normal samples. Multivariate cox analysis demonstrated that the risk score of this signature, T stage and AFP are three independent risk indicators for HCC early recurrence in this external cohort. An integrated model by combining this signature, T stage and AFP showed great prognostic potential in predicting HCC early recurrence in this external cohort. Additionally, the 4 lncRNAs involved in this signature have also been previously studied in HCC or other diseases. For instance, AF131217.1 was reported as a fluid shear force-sensitive RNA, which plays a protective role in atherosclerosis process [88]. AC108463.1 is related to gastric cancer progression [89]. CMB9-22P13.1 participates in the development of various cancer types including lung squamous cell carcinoma, breast cancer and hepatocellular carcinoma [90–92]. A recent study indicated that CMB9-22P13.1 could upregulate HOTTIP and activate HIF-1α/VEGF signaling, leading to enhanced hepatocellular carcinoma progression and angiogenesis [93]. The increase of TMCC1-AS1 facilitates proliferation, migration, invasion and EMT of HCC cells, resulting in poor outcome of liver cancer patients [94]. Given that the 4 lncRNAs have been selected to construct a prognostic signature for predicting HCC early recurrence, their roles in HCC progression should be intensively investigated. A variety of lncRNA database and developed computational models could be applied for lncRNA function and lncRNA-disease association prediction [9, 10, 16, 17], which may be further validated by experiments.

In conclusion, we developed a 4-lncRNA signature for predicting early recurrence in HCC. The integrated model of the 4-lncRNA signature risk score with TNM and AFP presents great prognostic performance for predicting HCC early recurrence. This signature might provide novel prognostic and therapeutic biomarkers for HCC and act to be a potential drug predictor.

## Electronic supplementary material

Below is the link to the electronic supplementary material.

**Additional file 1. Figure S1.** Flowchart of our analysis strategy. **Figure S2.** Analyses of differentially expressed lncRNAs between the training group (N = 157) and normal (N = 50). **Figure S3.** HCC early recurrence analyses.

**Additional file 2. Table S1.** Clinical characteristic of 314 TCGA-LIHC patients. **Table S2.** Differentially expressed lncRNAs associated with DFS. **Table S3.** Primers used in RT qPCR. **Table S4.** Drugs respond differently in the low- and high-risk group HCC patients. **Table S5.** Clinical characteristic of 24 HCC patients from Jinling cohort.

## Data Availability

The dataset supporting the conclusions of this article is available in the TCGA-LIHC repository, http://cancergenome.nih.gov/.

## List of abbreviations

HCC: Hepatocellular carcinoma
lncRNA: Long non-coding RNA
TCGA-LIHC: The Cancer Genome Atlas Liver Hepatocellular Carcinoma
OS: overall survival
DFS: disease free survival
DElncs: differentially expressed lncRNAs
DEG: Differentially expressed gene
LASSO: least absolute shrinkage and selection operator
SVM-RFE: Support Vector Machine Recursive Feature Elimination
RS: risk score
ROC: Receiver Operating Characteristic
KEGG: Kyoto Encyclopedia of Genes and Genomes
GO: Gene Ontology
GSEA: Gene Set Enrichment Analysis
BP: biological pathways
CC: cellular components
MF: molecular functions
ssGSEA: Single sample Gene Set Enrichment Analysis
NES: normalized enrichment score
TIDE: Tumor Immune Dysfunction and Exclusion
GDSC: Genomics of Drug Sensitivity in Cancer
