# Supplementary material for: Combining a machine-learning derived 4-lncRNA signature with AFP and TNM stages in predicting early recurrence of hepatocellular carcinoma
Source: BMC Genomics. 2023 Feb 27;24:89. doi: 10.1186/s12864-023-09194-8 (PMC9972730; doi:10.1186/s12864-023-09194-8)
Supplement: Supplementary file 1 — Additional file 1. Figure S1. Flowchart of our analysis strategy. Figure S2. Analyses of differentially expressed lncRNAs between the training group (N = 157) and normal (N = 50). Figure S3. HCC early recurrence analyses. [file 12864_2023_9194_MOESM1_ESM.pdf]

SUPPLEMENTARY FILES

Figures and Figure Legends

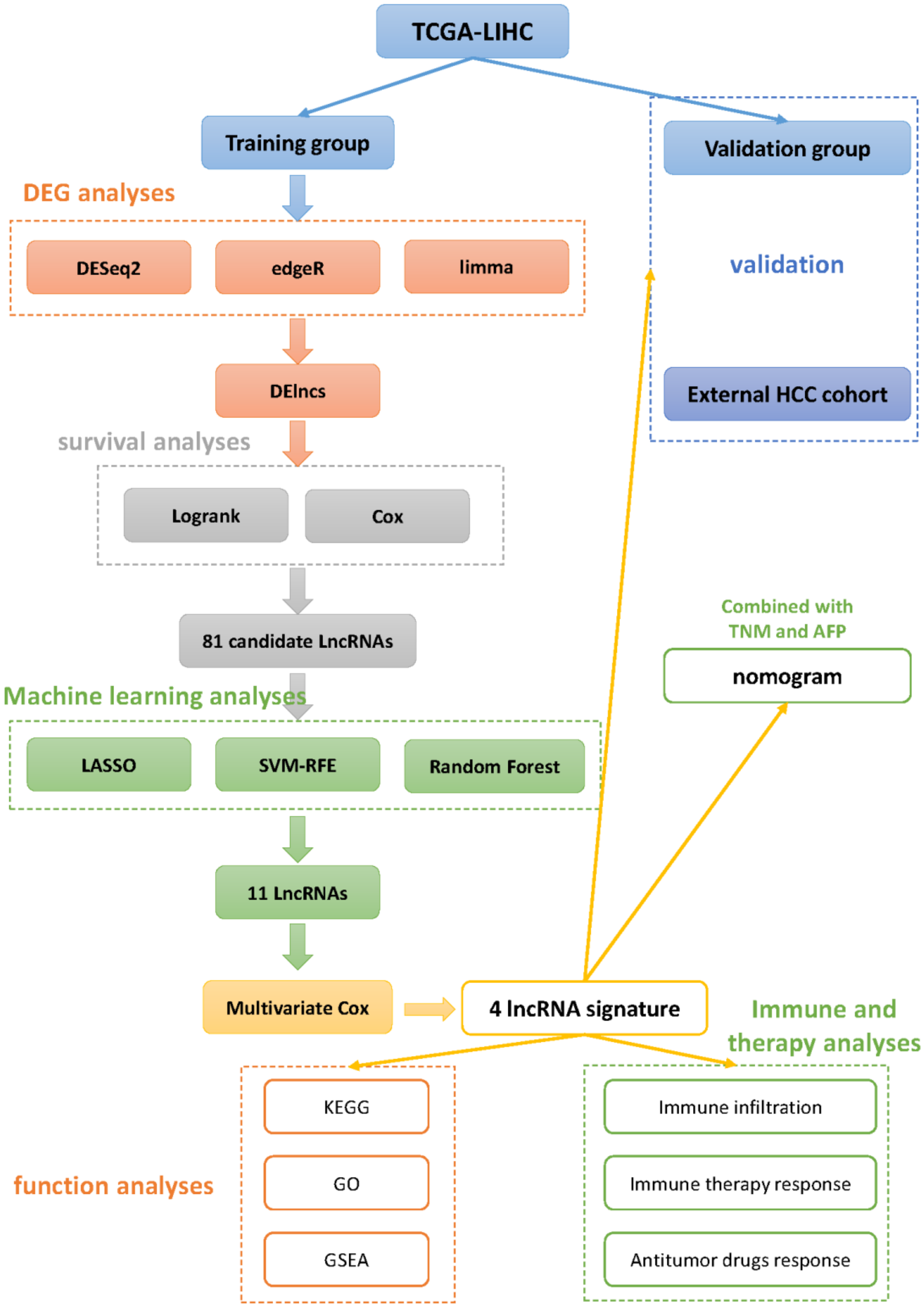

Figure S1 Flowchart of our analysis strategy

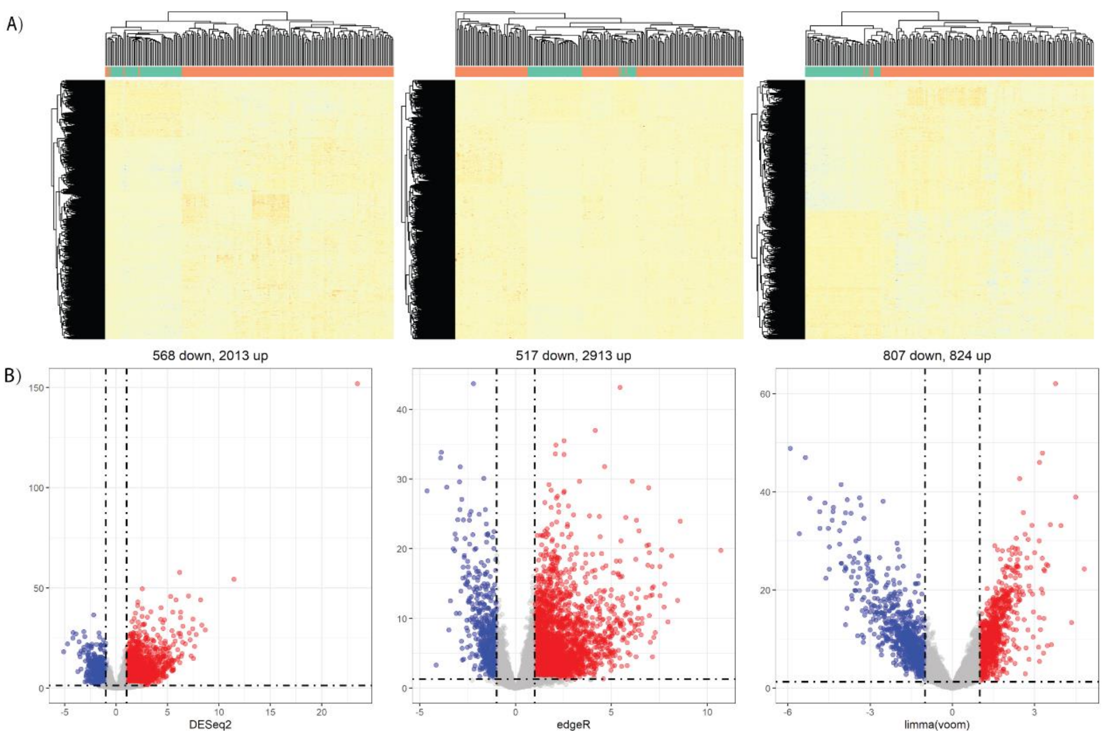

Figure S2 Analyses of differentially expressed lncRNAs between the training group (N = 157) and normal (N = 50)

A) Heatmap of differentially expressed lncRNAs from DESeq2, edgeR and limma; B) Volcano plot of 10795 lncRNAs from DESeq2 (2013 upregulated and 568 downregulated), edgeR (2913 upregulated and 517 downregulated) and limma (824 upregulated and 807 downregulated). Log|FC| > 1, P < 0.05.

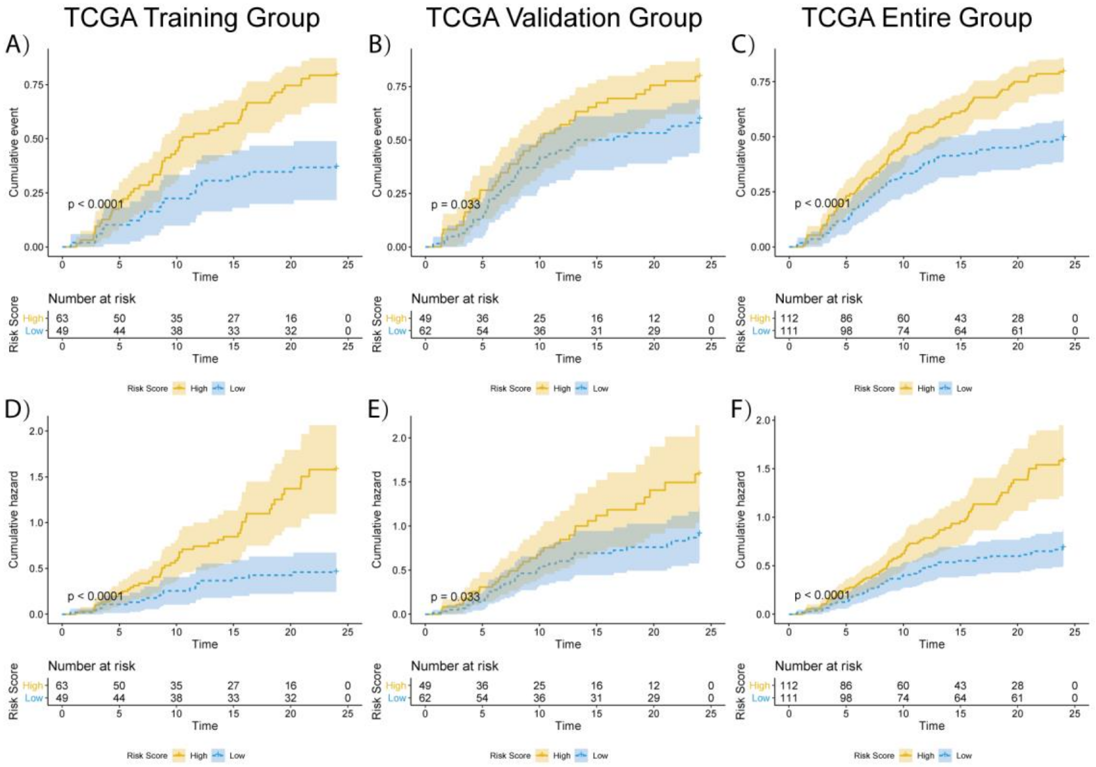

Figure S3 HCC early recurrence analyses

A) - C) Cumulative event probability curve between the low- and high-risk group in A) TCGA training group (N = 112, 49 and 63 in the low- and high-risk group), B) TCGA validation group (N = 111, 62 and 49 in the low- and high-risk group), C) TCGA entire group (N = 223, 111 and 112 in the low- and high-risk group), the cumulative event probabilitieswere significantly higher in the high-risk group compared with those in the low-risk group (A: P < 0.0001, B: P =0.033, C: P< 0.001); D) - F) Cumulative hazard curve between the low- and high-risk group in D) TCGA training group (N = 112, 49 and 63 in the low- and high-risk group), E) TCGA validation group (N = 111, 62 and 49 in the low- and high-risk group), F) TCGA entire group (N = 223, 111 and 112 in the low- and high-risk group), the cumulative hazard was significantly higher in the high-risk group compared with those in the low-risk group (A: P < 0.0001, B: P =0.033, C: P< 0.001).
